# High pathogenicity and low genetic evolution of avian paramyxovirus type I (Newcastle disease virus) isolated from live bird markets in Uganda

**DOI:** 10.1186/1743-422X-11-173

**Published:** 2014-10-01

**Authors:** Denis K Byarugaba, Kizito K Mugimba, John B Omony, Martin Okitwi, Agnes Wanyana, Maxwell O Otim, Halid Kirunda, Jessica L Nakavuma, Angélique Teillaud, Mathilde C Paul, Mariette F Ducatez

**Affiliations:** College of Veterinary Medicine, Makerere University, P.O. Box 7062, Kampala, Uganda; Uganda National Council of Science and Technology, P.O. Box 6884, Kampala, Uganda; National Livestock Resources Research institute, P.O. Box 96, Tororo, Uganda; INRA UMR 1225 IHAP, F-31076 Toulouse, France; Université de Toulouse, INP, ENVT, UMR 1225, IHAP, F-31076 Toulouse, France

**Keywords:** Newcastle disease virus, Live bird markets, Genetic diversity, Pathogenicity, Uganda

## Abstract

**Background:**

Newcastle disease is still a serious disease of poultry especially in backyard free-range production systems despite the availability of cross protective vaccines. Healthy-looking poultry from live bird markets have been suspected as a major source of disease spread although limited studies have been conducted to ascertain the presence of the virulent strains in the markets and to understand how they are related to outbreak strains.

**Methods:**

This study evaluated the occurrence of Newcastle disease virus in samples collected from poultry in live bird markets across Uganda. The isolates were pathoyped using standard methods (mean death time (MDT), intracelebral pathogenicity index (ICPI), and sequencing of the fusion protein cleavage site motif) and also phylogenetically analysed after sequencing of the full fusion and hemagglutin-neuraminidase genes. The isolates were classified into genotypes and subgenotypes based on the full fusion protein gene classification system and compared with other strains in the region and world-wide.

**Results:**

Virulent avian paramyxovirus type I (APMV-1) (Newcastle disease virus) was isolated in healthy-looking poultry in live bird markets. The viruses belonged to a new subgenotype, Vd, in genotype V, and clustered together with Tanzania and Kenya strains. They harbored low genetic diversity.

**Conclusion:**

The occurrence of virulent AMPV-1 strains in live bird markets may serve as sources of Newcastle disease outbreaks in non-commercial farms.

## Background

Newcastle disease is a highly contagious disease affecting chickens and other poultry species and wild birds. It devastates unvaccinated flocks in periodic outbreaks resulting in up to 70-100% mortality, which is a big loss to the households. It is caused by RNA viruses of the Avian Paramyxovirus type 1 (APMV-1), (synonym: Newcastle disease virus, NDV) [1]. Based on clinical signs, these viruses have been grouped by virulence phenotypes: asymptomatic, lentogenic, mesogenic, and velogenic strains reflecting increasing levels of virulence. Velogens, the most virulent viruses, are those that may cause extensive hemorrhagic lesions, particularly in the gastrointestinal tract (viscerotropic), and/or a predominance of nervous signs (neurotropic) as well as respiratory signs. The lentogenic strains are common among domestic poultry and wild bird populations with insignificant clinical symptoms.

Although the disease is endemic in most African countries, there are still limited studies regarding the ecology, molecular epidemiology, genetic diversity, and distribution of NDV on the continent, not to mention the gap in field vaccine efficacy studies. Most research has focused mainly on basic epidemiology especially during ND outbreaks in poultry and it is believed ND in village chickens is attributed to birds that are shedding virus during and after incubation or post vaccination [2, 3]. It is thought that while vaccination confers protection, some birds continue shedding the virus while appearing healthy. It has been suggested that outbreaks in the dry season are not due to better virus survival under these conditions but to higher trading of birds during this period [4]. Markets may serve as a source of Newcastle disease infection and contribute to its spread as infected birds not showing clinical signs are sold before they come down with the disease.

Genetically, NDV strains are broadly divided into avirulent and virulent subtypes on the basis of the precursor fusion (F) protein cleavage activation site that must be cleaved into F1 and F2 in order for the virus to be infectious. The virulent strains have more basic amino acids than their avirulent counterparts: 112(R/K)RQ(R/K)R*F117 and 112(G/E)(K/R)Q(G/E)R*L117 motifs at the cleavage site for virulent and avirulent viruses, respectively [5]. Biologically the virulent forms by international agreement for purposes of trade [5] are determined with intracerebral pathogenicity indices (ICPI) of ≥ 0.7 but they can also be evaluated by mean death time (MDT) and intravenous pathogenicity index (IVPI) methods.

The genetic classification of the APMV-1 continues to evolve. The simultaneous use of two systems for genotyping APMV-1 created some confusion and lineages and genotypes classification have been reported under different systems. Aldous et al. described a clustering method for the APMV-1 based on the precursor fusion protein cleavage activation site [6]. Recently a more rational genotyping method was proposed by Diel et al. based on mean interpopulational evolutionary distances of the full F protein with cutoff values to assign new genotypes (>10% mean interpopulational evolutionary distance) and new sub-genotypes (3 to 10% mean interpopulational evolutionary distance) [7]. While both systems agree to class I viruses having a single lineage, the class II under Diel et al. contained 15 genetic groups including 10 previously established (I-IX, and XI) and five new genotypes (X, XII, XIII, XIV and XV) [7]. This new unified nomenclature system is expected to provide a clearer and more uniform classification system of NDV isolates and facilitate common understanding of the NDV epidemiology, evolution, disease control and diagnosis. The drawback is that it requires extra work to obtain the F gene full sequences.

Genotyping based on other APMV-1 genes still concur with the basic grouping into classes I and II with class II viruses comprising the vast majority of sequenced NDV. This includes most of the virus isolates recovered from poultry (gallinaceous birds) and from pet and wild birds [8]. Phylogenetic studies of both the F and the hemagglutinin-neuraminidase (HN) protein genes of NDV have been used for most molecular epidemiologic analyses and characterization of NDV into specific lineages [9] even prior to the proposed Diel at al. classification [7]. Restriction enzyme site mapping of the F protein gene and sequence analysis have also been used to classify NDV isolates into seven genotypes and was the basis for identification of a novel genotype of NDV isolates from Uganda reported in 2004 [10]. Different lineages have been described in many parts of Africa basing on the fusion protein cleavage site [6] and a few are beginning to use the system described by Diel et al., [7, 11, 12]. Snoeck et al. thus recently completed the classification by Diel et al. [7] on the same criteria and showed that 17 NDV class II genotypes (named I to XVIII but with no genotype XV) co-circulate worldwide [11]. The purpose of this study was to assess the occurrence of NDV pathotypes in live bird markets across Uganda and to determine their genetic diversity and relatedness using the new NDV nomenclature.

## Results and discussion

**Figure 1:**
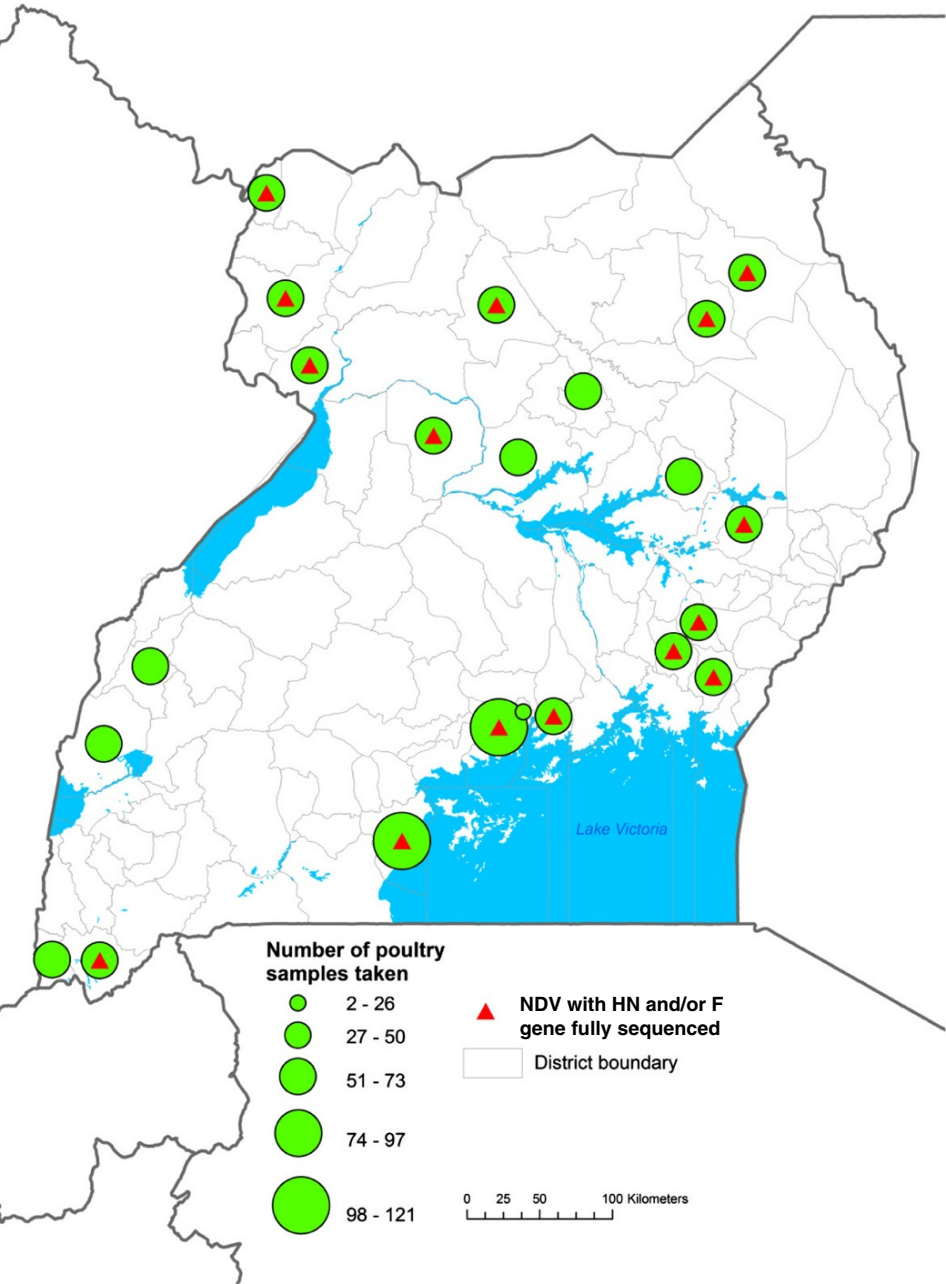
**Areas in Uganda (East Africa) where samples were collected.** The map shows the distribution of representative isolates that were sequenced throughout the country. The green spheres show area of origin and number of samples while the red triangles show where sequence data originated.

In total, 1,357 samples were collected from which 114 isolates were recovered (8.7% prevalence by isolation) during the study period and only 19 paired samples yielded virus from both tracheal and cloacal swabs. The isolates were recovered in almost all the markets across the country (Figure 1). The northern region had the highest isolation rate of 22.1%, followed by central region at 13.1%, then the eastern region at 11.2% and the lowest prevalence was in the western region at 6.9%.

Most of these markets sold chickens and indeed most of the isolates (109 isolates from 1250 chicken samples; 8.7% isolation rate) were from chickens. Only two isolates were obtained from thirty four turkeys samples (5.8%) (NDV/turkey/Uganda/MU041/2011 and NDV/turkey/Uganda/MU042/2011) and three isolates from seventy three ducks samples (5.2%) (NDV/duck/Uganda/MU062/2011, NDV/duck/Uganda/MU078/2011 and NDV/duck/Uganda/MU079/2011). None of the turkeys or ducks was showing any signs of disease at the time of sample collection, while some of the chickens were showing symptoms of ND at the time of sample collection with an isolation rate of 28.6% (6/21) and 9.0% (108/1229) from sick and healthy chickens respectively.

### Biological characterization of isolates

Intracelebral pathogenicity indices (ICPI) and mean death time (MDT) methods demonstrated that all the isolates were virulent with variations in virulence. According to the values of 112 isolates on which MDT was performed, most of the isolates (85%: 95/112) were velogenic while the remaining (15%: 17/112) were mesogenic (only data values for isolates for which full sequences for either F or HN or both genes are shown in Table 1, the rest of the data is not shown). According to ICPI values, all the isolates had very high values ranging from 1.60- 1.86 (except isolate NDV/chicken/Uganda/MU013/2011 with ICPI of 0.35) indicating that they were highly virulent. The isolates were further assessed by examining the amino acids sequences of the fusion protein (F0) at position 112–117 at the cleavage site and all had a “^112^RRQKR*F^117^” motif which confirmed them as virulent (Table 1).

**Table 1:** **Biological characteristics of NDV isolated in this study**

Isolate number	District of origin	MDT (hrs)	ICPI	F0 cleavage site motif (Pos: 111–118)	Accession # for full HN gene	Accession # for full F gene	Pathotype
NDV/chicken/Uganda/MU001/2011	MASAKA	46.2	1.75	GRRQKR’FV	HG937535	HG937567	V
NDV/chicken/Uganda/MU004/2011	MASAKA	46.4	1.76	GRRQKR’FV	^1^	^2^	V
NDV/chicken/Uganda/MU005/2011	MASAKA	47.8	1.77	GRRQKR’FV	^1^	^2^	V
NDV/chicken/Uganda/MU007/2011	MASAKA	46	1.76	GRRQKR’FV	HG937536	HG937568	V
NDV/chicken/Uganda/MU009/2011	MASAKA	48	1.78	GRRQKR’FV	HG937537	HG937569	V
NDV/chicken/Uganda/MU010/2011	MASAKA	46.8	1.79	GRRQKR’FV	HG937538	HG937570	V
NDV/chicken/Uganda/MU013/2011^+^	MUKONO	80.1	0.35	GRRQKR’FV	HG937539	HG937571	M
NDV/chicken/Uganda/MU014/2011*	MUKONO	54.2	1.77	GRRQKR’FV	HG937540		V
NDV/chicken/Uganda/MU019/2011	WAKISO	68.8	1.68	GRRQKR’FV	HG937541	HG937572	M
NDV/chicken/Uganda/MU022/2011*	ABIM	44.8	1.77	GRRQKR’FV	HG937542		V
NDV/chicken/Uganda/MU024/2011	ABIM	ND	ND	GRRQKR’FV		HG937573	
NDV/chicken/Uganda/MU026/2011	BUGIRI	74.5	1.61	GRRQKR’FV	HG937543	HG937574	M
NDV/chicken/Uganda/MU028/2011	BUGIRI	40.2	1.79	GRRQKR’FV	^3^	^4^	V
NDV/chicken/Uganda/MU029/2011*	BUGIRI	40.1	1.78	GRRQKR’FV	HG937544		V
NDV/chicken/Uganda/MU030/2011*	IGANGA	36.8	1.78	GRRQKR’FV	HG937545		V
NDV/chicken/Uganda/MU031/2011*	IGANGA	46.9	1.78	GRRQKR’FV	HG937546		V
NDV/chicken/Uganda/MU032/2011	IGANGA	36.8	1.77	GRRQKR’FV		HG937575	V
NDV/chicken/Uganda/MU033/2011	IGANGA	40.2	1.78	GRRQKR’FV		HG937576	V
NDV/chicken/Uganda/MU035/2011	IGANGA	44.2	1.75	GRRQKR’FV	HG937547	HG937577	V
NDV/chicken/Uganda/MU037/2011	KOTIDO	68.2	1.7	GRRQKR’FV		HG937578	M
NDV/chicken/Uganda/MU038/2011*	KOTIDO	71.2	1.69	GRRQKR’FV			M
NDV/chicken/Uganda/MU039/2011	KUMI	48.6	1.7	GRRQKR’FV	HG937548	HG937579	V
NDV/chicken/Uganda/MU040/2011	KUMI	58.9	ND	GRRQKR’FV		HG937580	V
NDV/chicken/Uganda/MU044/2011	NAMUTUMBA	56.8	1.66	GRRQKR’FV		HG937581	V
NDV/chicken/Uganda/MU050/2011	ARUA	50.6	1.75	GRRQKR’FV		HG937582	V
NDV/chicken/Uganda/MU052/2011*	ARUA	38.4	1.8	GRRQKR’FV	HG937550		V
NDV/chicken/Uganda/MU054/2011*	ARUA	38.4	1.82	GRRQKR’FV	HG937551		V
NDV/chicken/Uganda/MU056/2011	ARUA	54.4	1.67	GRRQKR’FV	HG937552	HG937583	V
NDV/chicken/Uganda/MU058/2011*	ARUA	34.6	1.78	GRRQKR’FV	HG937535		V
NDV/chicken/Uganda/MU059/2011*	ARUA	48.2	1.76	GRRQKR’FV	HG937553		V
NDV/duck/Uganda/MU062/2011	ARUA	58	1.74	GRRQKR’FV		HG937584	V
NDV/chicken/Uganda/MU063/2011	ARUA	48.6	1.76	GRRQKR’FV		^5^	V
NDV/chicken/Uganda/MU069/2011	KIRYANDONGO	42.3	1.79	GRRQKR’FV	HG937554	HG937585	V
NDV/chicken/Uganda/MU071/2011	KIRYANDONGO	48.8	1.75	GRRQKR’FV	HG937555	HG937586	V
NDV/chicken/Uganda/MU072/2011*	KIRYANDONGO	38.2	1.79	GRRQKR’FV	HG937556		V
NDV/chicken/Uganda/MU074/2011	KOBOKO	48.2	1.77	GRRQKR’FV	HG937557	HG937587	V
NDV/chicken/Uganda/MU077/2011*	KOBOKO	51.1	1.75	GRRQKR’FV	HG937558		V
NDV/chicken/Uganda/MU083/2011*	NEBBI	48.2	1.76	GRRQKR’FV	HG937559		V
NDV/chicken/Uganda/MU084/2011	NEBBI	64.2	1.62	GRRQKR’FV	HG937560	HG937588	M
NDV/chicken/Uganda/MU090/2011	GULU	46.2	1.77	GRRQKR’FV	HG937561	HG9375689	V
NDV/chicken/Uganda/MU091/2011*	GULU	44.3	1.83	GRRQKR’FV	HG937562		V
NDV/chicken/Uganda/MU096/2011*	GULU	56.5	ND	GRRQKR’FV	HG937563		V
NDV/chicken/Uganda/MU098/2011*	GULU	44.2	1.77	GRRQKR’FV	HG937564		V
NDV/chicken/Uganda/MU099/2011*	GULU	50.2	1.75	GRRQKR’FV	^6^		V
NDV/chicken/Uganda/MU102/2011*	GULU	37.6	1.79	GRRQKR’FV	^7^		V
NDV/chicken/Uganda/MU105/2011*	GULU	55.7	1.74	GRRQKR’FV	^7^		V
NDV/chicken/Uganda/MU108/2011*	KABALE	49.8	1.72	GRRQKR’FV	HG937565		V
NDV/chicken/Uganda/MU111/2011	KASESE	52.1	1.86	GRRQKR’FV	HG937566	HG937590	V
NDV/chicken/Uganda/MU113/2011	KASESE	56.4	ND	GRRQKR’FV		HG937591	V

The table presents isolates for which full HN or F genes or both were sequenced. ND = Not done; V = velogenic; M = mesogenic; the pathotype was based on MDT values as described in materials and methods; *only partial F gene sequences were obtained for these isolates with FIP1-FIP2 primers targeting the F cleavage site area(Kho, Mohd-Azmi et al. [32]). ^1^HN gene sequences identical to HN of NDV/chicken/Uganda/MU001/2011 (accession number: HG937535); ^2^ F gene sequences identical to F of NDV/chicken/Uganda/MU001/2011 (accession number: HG937567); ^3^HN gene sequences identical to HN of NDV/chicken/Uganda/MU026/2011 (accession number: HG937543); ^4^ F gene sequences identical to F of NDV/chicken/Uganda/MU026/2011(accession number: HG937574); F gene sequence identical to F of NDV/duck/Uganda/MU062/2011 (accession number: HG937584); HN gene sequences identical to HN of NDV/chicken/Uganda/MU096/2011 (accession number: HG937563); ^7^HN gene sequences identical to HN of NDV/chicken/Uganda/MU098/2011 (accession number: HG937564). ^+^MDT and ICPI were performed twice for NDV/chicken/Uganda/MU013/2011 with little difference; the average values shown in the Table.

### Phylogenetics analysis of Ugandan NDV isolates

**Figure 2:**
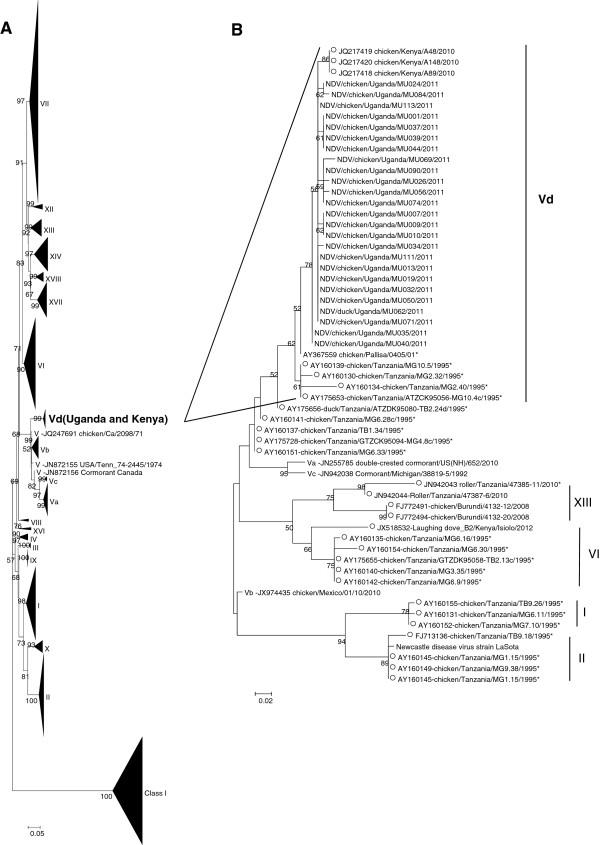
**Phylogenetic trees (ML trees) of the fusion (F) gene of Ugandan Newcastle disease virus (NDV) isolates at the nucleotide level.** Partial F gene sequences are indicated with a “*” symbol after the strain name. A single representative virus was selected for strains with identical nucleotide sequences: NDV/chicken/Uganda/MU001/2011 was identical to NDV/chicken/Uganda/MU004/2011 and NDV/chicken/Uganda/MU005/2011; NDV/chicken/Uganda/MU026/2011 to NDV/chicken/Uganda/MU028/2011; and NDV/duck/Uganda/MU062/2011 to NDV/chicken/Uganda/MU063/2011. The F sequences of our Ugandan NDV isolates were compared with **A)** all full F gene sequences available in GenBank database: 1209 sequences, and **B)** the reference vaccine strain Lasota, a representative strain for each of the genotype V subgenotypes, and all the East African NDV strains available in the database (indicated with an open circle).

**Figure 3:**
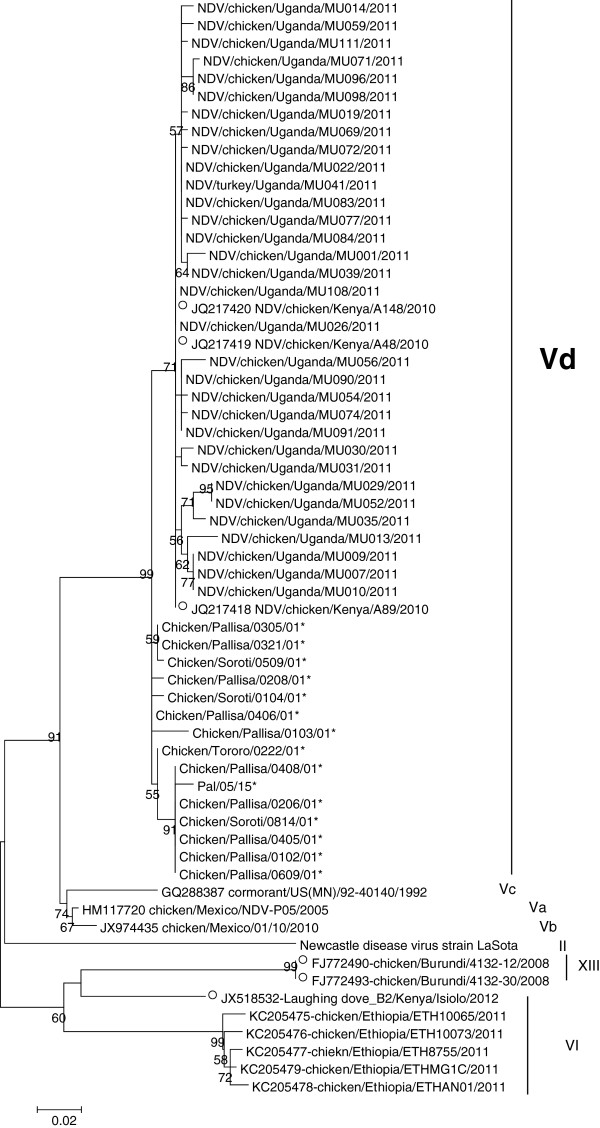
**Phylogenetic tree (ML tree) of the hemagglutinin-neuraminidase (HN) gene of Ugandan Newcastle disease virus (NDV) isolates at the nucleotide level.** The HN sequences of our Ugandan NDV isolates were compared with relevant virus sequences available in GenBank database: the reference vaccine strain Lasota, all the East African NDV strains available in the database (indicated with an open circle), Ethiopian strains, and a representative strain for each of the genotype V subgenotypes. Partial HN gene sequences are indicated with a “*” symbol after the strain name. A single representative virus was selected for strains with identical nucleotide sequences: NDV/chicken/Uganda/MU001/2011 was identical to NDV/chicken/Uganda/MU004/2011 and NDV/chicken/Uganda/MU005/2011; NDV/chicken/Uganda/MU007/2011 to NDV/chicken/Uganda/MU009/2011; NDV/chicken/Uganda/MU026/2011 to NDV/chicken/Uganda/MU028/2011; NDV/chicken/Uganda/MU059/2011 to NDV/duck/Uganda/MU062/2011 and NDV/chicken/Uganda/MU063/2011; NDV/chicken/Uganda/MU096/2011 to NDV/chicken/Uganda/MU099/2011 and NDV/chicken/Uganda/MU102/2011; and NDV/chicken/Uganda/MU098/2011 to NDV/chicken/Uganda/MU105/2011.

A total of 49 representative isolates were fully sequenced for either F (29 isolates) or HN (38 isolates) genes. Of these, 19 isolates were sequenced for both F and HN genes. All the 49 isolates were sequenced for the cleavage site of the F gene and all harbored a multibasic “^112^RRQKR*F^117^” motif at the site, suggesting a velogenic phenotype. These isolates also had a valine amino acid (aa) at position 118, and clustered with genotype V viruses [7, 11] which corresponds to lineage 3c according to Aldous et al. [6] and the few previously available Ugandan NDV sequences for both their F (Figure 2) and HN (Figure 3) genes.

To complete the recent efforts on NDV classification with the criteria defined by Diel et al. [7], we aimed at (i) confirming the robustness of genotype V after the addition of new F gene sequences, and (ii) redefining sub-genotypes within genotype V. As shown on Figure 2a and 2b, genotype V was clearly supported by the ML tree topology and by a high bootstrap value (99, Figure 2a). Phylogenetic analyses carried out on the basis of the first 375 nucleotides of the F gene confirmed the strains genotypes (data not shown). Table 2 summarizes the evolutionary distances between class II NDV genotypes. All evolutionary distances between genotype V sequences and their class II NDV counterparts (numbers in bold in Table 2) were >10%, confirming the robustness of genotype V. Within genotype V, the East African viruses seemed to cluster separately from sub-genotypes Va, Vb, and Vc viruses (Figure 2a, node supported by a bootstrap value of 99). The ML tree findings were confirmed by evolutionary distances comparisons: distances between the subgenotype Vd East African NDV viruses included in the analysis and Va, Vb and Vc sub-genotypes ranged between 10 and 13% (Table 3), which is higher than the distance required for a new sub-genotype to be defined (3 to 10%, [7]). The analysis of evolutionary distance between genotypes was carried out considering the sub-genotype Vd as a full genotype and showed distances ranging between 11.3 and 23.9% between Vd viruses and the 17 other genotypes (11.3% between Vd and V; 23.9% between Vd and XI, data not shown). However it seems difficult to consider East African NDV viruses (Vd) as a distinct full genotype when looking at the tree topography only.

**Table 2:** **Estimates of evolutionary distances (in number of substitutions per site) over sequence pairs between genotypes of class II**

Genotype (number of sequences analyzed)	I	II	III	IV	IX	V	VI	VII	XII	VIII	X	XI	XIII	XIV	XVI	XVII	XVIII
I (n = 113)		*0.009*	*0.009*	*0.008*	*0.008*	*0.011*	*0.010*	*0.012*	*0.010*	*0.008*	*0.013*	*0.011*	*0.011*	*0.013*	*0.011*	*0.012*	*0.012*
II (n = 128)	0.118		*0.010*	*0.010*	*0.010*	*0.013*	*0.012*	*0.014*	*0.011*	*0.009*	*0.015*	*0.014*	*0.013*	*0.017*	*0.013*	*0.014*	*0.013*
III (n = 9)	0.103	0.118		*0.008*	*0.009*	*0.011*	*0.010*	*0.012*	*0.010*	*0.010*	*0.013*	*0.012*	*0.011*	*0.014*	*0.011*	*0.013*	*0.011*
IV (n = 6)	0.101	0.115	0.074		*0.008*	*0.010*	*0.009*	*0.011*	*0.008*	*0.010*	*0.010*	*0.011*	*0.010*	*0.012*	*0.009*	*0.011*	*0.011*
IX (n = 25)	0.104	0.120	0.081	0.073		*0.011*	*0.010*	*0.012*	*0.010*	*0.010*	*0.012*	*0.012*	*0.011*	*0.014*	*0.011*	*0.012*	*0.011*
**V (n = 124)**	**0.179**	**0.193**	**0.163**	**0.140**	**0.161**		*0.009*	*0.010*	*0.009*	*0.013*	*0.014*	*0.011*	*0.010*	*0.012*	*0.010*	*0.011*	*0.010*
VI (n = 139)	0.161	0.179	0.149	0.120	0.148	**0.144**		*0.009*	*0.008*	*0.011*	*0.013*	*0.008*	*0.008*	*0.011*	*0.009*	*0.010*	*0.009*
VII (n = 312)	0.170	0.195	0.154	0.133	0.157	**0.158**	0.124		*0.010*	*0.013*	*0.015*	*0.009*	*0.009*	*0.011*	*0.011*	*0.010*	*0.009*
XII (n = 8)	0.139	0.154	0.125	0.097	0.121	**0.131**	0.106	0.119		*0.011*	*0.013*	*0.009*	*0.008*	*0.011*	*0.009*	*0.010*	*0.010*
VIII (n = 4)	0.104	0.109	0.118	0.114	0.114	**0.185**	0.166	0.175	0.151		*0.014*	*0.013*	*0.012*	*0.015*	*0.012*	*0.013*	*0.013*
X (n = 19)	0.177	0.188	0.161	0.115	0.151	**0.206**	0.197	0.212	0.181	0.185		*0.015*	*0.014*	*0.017*	*0.013*	*0.015*	*0.015*
XI (n = 4)	0.171	0.193	0.154	0.135	0.155	**0.160**	0.114	0.103	0.114	0.173	0.211		*0.007*	*0.010*	*0.011*	*0.008*	*0.008*
XIII (n = 27)	0.167	0.189	0.154	0.129	0.146	**0.154**	0.121	0.105	0.115	0.173	0.203	0.096		*0.009*	*0.010*	*0.008*	*0.007*
XIV (n = 52)	0.203	0.234	0.197	0.170	0.196	**0.184**	0.150	0.140	0.151	0.208	0.250	0.126	0.126		*0.013*	*0.010*	*0.010*
XVI (n = 4)	0.151	0.172	0.142	0.111	0.139	**0.152**	0.138	0.154	0.118	0.157	0.191	0.150	0.141	0.180		*0.012*	*0.011*
XVII (n = 54)	0.171	0.202	0.172	0.149	0.162	**0.161**	0.136	0.127	0.134	0.182	0.216	0.114	0.112	0.127	0.164		*0.008*
XVIII (n = 15)	0.176	0.189	0.163	0.145	0.155	**0.159**	0.122	0.119	0.127	0.178	0.208	0.104	0.101	0.125	0.159	0.104	

The number of base substitutions per site from averaging over all sequence pairs between groups are shown. Standard error estimate(s) are shown above the diagonal, in italic font. Analyses were conducted using the Maximum Composite Likelihood model. The rate variation among sites was modeled with a gamma distribution (shape parameter = 1). All positions containing gaps and missing data were eliminated. Numbers in boldface refer to genotype V viruses.

**Table 3:** **Estimates of evolutionary distances (in number of substitutions per site) over sequence pairs between sub-genotypes V**

Genotype (number of sequences analyzed)	Vd	Va	Vb	Vc
**Vd (n = 25)**		*0.011*	*0.008*	*0.010*
Va (n = 45)	**0.128**		*0.007*	*0.006*
Vb (n = 33)	**0.101**	0.101		*0.007*
Vc (n = 5)	**0.118**	0.066	0.093	

The number of base substitutions per site from averaging over all sequence pairs between groups are shown. Standard error estimate(s) are shown above the diagonal, in italic font. Analyses were conducted using the Maximum Composite Likelihood model. The rate variation among sites was modeled with a gamma distribution (shape parameter = 1). All positions containing gaps and missing data were eliminated. Numbers in boldface refer to sub-genotype Vd.

We also compared the Uganda strains with the genotype Vd viruses from East Africa. The Ugandan strains could only be differentiated from the Kenyan strains by substitutions in the HN protein by only two amino acids Y205F and A546V. However there were residues shared by Kenyan and Ugandan strains at positions S440N and T48A: half of the Uganda isolates shared the same amino acids S440 and T48 with Kenyan isolates. The F-protein did not have any specific residues that differentiated between the Ugandan and other genotype Vd East Africa strains.

In an attempt to estimate whether the current vaccine strain in use in Uganda could be effectively inducing protective antibodies against the circulating field strains, we compared the major neutralizing antibody epitopes previously reported [11, 12] within the F and HN proteins between the Lasota vaccine strain and our field isolates from this study. This revealed that all the Ugandan and indeed the East Africa Vd isolates shared similar residues D72, E74, A75, K78, A79, L343 and ^151^ILRLKESIAATNEAVHEVTDG^171^ in the F-protein with the Lasota vaccine strain. In the HN protein, both the Uganda strains and the Lasota vaccine strain also shared similar neutralizing antibody epitopes residues; D287, E347, D349, Y350, R353, K356, R513, I514 (except for 2 strains), S519, S521 and N569. The neutralizing epitopes of the Ugandan strains however differed from the Lasota strain at some residues. The Uganda strains had R333 (except one strain-MU069) and N569 while the Lasota had K333 and D569.

### Genetic diversity of NDV in Uganda

Low genetic diversity was observed among 2011 Ugandan NDV strains with Kimura distances ranging from 0.0 to 2.6% and from 0.0 to 3.5% for the F and HN genes, respectively; but also among known Ugandan NDV strains (virus sequences available from 2001 and 2011 only) with Kimura distances (on partial genes sequences) ranging from 1.5 to 2.6% (comparison between our 2011 sequences and the partial F gene sequence of NDV/chicken/Pallisa/0405/2001) and from 1.6 to 4.1% (comparison between our 2011 sequences and the partial HN gene sequence of 15 partial Ugandan 2001 NDV sequences) for the F and HN genes, respectively.

Our study isolated from apparently health poultry in live bird markets ND viruses that were velogenic in eggs and chickens and harbored polybasic F-protein cleavage site ^112^RRQKR’F^117^ amino acids. This was further confirmed by the ICPI which revealed that all the isolates for which ICPI was done had values close to 2.0 (1.60-1.86) with only one isolate (NDV/chicken/Uganda/MU013/2011) with a value of 0.35. The high MDT value and the cleavage site motif of NDV/chicken/Uganda/MU013/2011 however indicated that it was also virulent. Although fairly accurate and sensitive there could be some possible differences in ICPI results related to the execution or final dose that reaches the target site as previously described [13]. The variations in virulence of ND viruses are well known and although the use of MDT provides well defined cut-off values for classification for grouping the viruses into lentogenic, mesogenic and velogenic strains, it has been indicated to be imprecise and unreliable and OIE recommends use of ICPI indices above 0.7 by international agreement for purposes of ND outbreak notification [5].

Traditionally, non-commercial poultry farmers have recognized that introduction of birds from live bird markets was followed by Newcastle disease outbreaks. It is only recently that virological studies showing that healthy-looking birds in live bird markets carry virulent ND viruses have come through to confirm this long known belief [14, 15]. While more and more genetic studies of Newcastle disease are coming out from Africa, many of them have been limited in number of isolates, extent of the sequences analysed, regional as well in-country representation.

A little more studies and genetic analysis of isolates from West Africa have been done. Diverse genotypes of NDV were reported in these studies [11, 14–19] complementing other studies in the rest of the world. In addition, genetic diversity at the aa level observed among Western African viruses by Snoeck et al. [11, 15] is much higher than what we observed in Uganda. More precisely, when we calculated the Kimura distance (at the nt level) for available complete F gene sequences from Nigeria for example (country in West Africa with most NDV sequence information) within a single genotype it ranged between 0 and 4.6% for genotype XIVa (strains from 2007 to 2011 n = 15; 0 to 4.6% for 2009 strains alone, n = 12) and between 0 and 5.8% for genotype XVIIa (strains from 2006 to 2011 n = 38; 0.1 to 5.0% for 2009 strains alone, n = 21). This difference could be because (i) there are very few isolates from East Africa that have been examined at the genetic level [10, 20, 21] and for most of them only partial sequences at the cleavage site of the fusion gene were examined but also because (ii) Nigeria is a much bigger country than Uganda, with more complex poultry trade with neighbors but also international trade. The Kimura distance we observed in Uganda was indeed <2.5% for the F gene between 2001 and 2011.

Until recently most of the classification of these viruses was based on the F-protein cleavage site partial sequences and their restriction analysis [3] but this method harbored inconsistencies for example in the evolutionary distances between genotypes and subgenotypes [20]. In the present study we used the new classification system proposed by Diel et al. [7] for nomenclature and classification of the class II ND viruses to classify our viruses and compare them with other East Africa strains with full F gene sequence available in GenBank. Our study confirmed the benefit of using this new classification system for NDV strains but at the same time highlighted some differences in the different genotyping criteria; specifically the distance estimates percentages and tree topology. In our current analysis, while most of the East African NDV strains have a sufficient distance estimate from the other genotype V strains to form a new genotype, the tree topology analysis does not differentiate them into a different genotype but rather groups them among genotype V viruses (Figure 2). This highlights the importance of both distance estimates and tree topology for NDV genotyping and suggests that criteria used for genotyping may need to be updated in the future.

The Ugandan viruses from our study clustered in genotype V with most other NDV sequences from Kenya and Tanzania that were available in GenBank for analysis. Interestingly, there was very limited diversity among our isolates, all of them clustering in genotype V. Our strains also differed from the genotype Ia strains identified in Tanzania (previously classified as lineage 1a) [20], genotype II strains identified in Tanzania and Ethiopia (previously classified as lineage 2) [20, 22], genotype IV strain identified in Sudan (previously classified as lineage 3b) [23], genotype XIII isolates from Burundi and Tanzania (previously classified as genotype VIIb or lineage 5b) [16, 20], genotype VIf strains from Tanzania and Sudan [20, 23], genotype VI isolates from Ethiopia (as determined by the phylogeny carried out in the present study), and genotype VIId isolates from Sudan and Ethiopia [22, 23] as well as South Africa strains [24] (previously classified as genotype VIId). The most common virulent genotypes known to cause disease across the world belonged to genotypes V, VI, VII and VIII [25]. Genotype V in which our isolates fall is said to have been most commonly isolated from Central and North America and only three subgenotypes (Va, Vb and Vc) have been described in the new classification while the most common West Africa strains belong to genotypes XIV, XVII and XVIII [11]. Our isolates did not cluster with the three subgenotypes according to the criteria proposed by Diel et al. [7], suggesting that they belonged to a new subgenotype which we have named subgenotype Vd.

The similarity or clustering of our isolates from the present study and some of the Kenyan and Tanzanian isolates might suggest cross border mixing of strains, which may occur through uncontrolled cross border trade of live birds carrying an endemic strain evolving within the Uganda-Kenya-Tanzania region. This would explain the difference between our isolates and the Sudan and Burundi strains which are different, suggesting limited mixing of the strains. The long time insurgency in Northern Uganda and South Sudan did not allow free trade along these borders and could explain this observation. While the difference with West Africa strains is geographically understandable, the limited number of isolates from countries in East Africa and unavailability of full F gene sequences could have limited fair comparison of the strains in the region. There could therefore be more diversity than we could ascribe with the limited strains sequences in GenBank. All our strains were defined by the “^112^RRQKR*FV^118^” motif at the cleavage site. These strains with valine at position 118 have been previously classified as lineage 3 [6] and this motif is common among most genotype V and have been reported in Madagascar [21] and other countries. However our isolates differ in this region where Madagascar isolates that had “^112^RR**RR**R*FV^118^” motif at the cleavage sites as compared to ours with RRQKR*FV motif, although all were isolated from apparently healthy non-vaccinated poultry. Previous studies have indicated that isolates with valine at position 118 results accompanied by substitution of the arginine by glutamine at position 114 resulted in reduction of viral replication and pathogenicity of the viruses [26]. Although no evaluation was done of the single I118V substitution without the Q114R substitution in that study, our isolates that contain the RRQKR*FV have been demonstrated to be highly virulent in experimental studies although they were isolated from apparently healthy poultry.

Genetic studies for NDV are not only important for diagnostic and pathogenicity assessment but are also becoming increasingly important in vaccine strain selection and vaccine studies. Reports have alluded to the possible challenges and implications for diagnosis based on genetic differences in the matrix gene supposed to be conserved across most strains and its implications for the current molecular diagnostic tools [16, 25]. More important is the implication for vaccination which is the current best option for controlling the disease. Several studies have demonstrated that while Newcastle disease virus or APMV-1 viruses belong to the same serotype, they have diverse genotypes that evolve independently and may impact vaccination success. It has been demonstrated that vaccination with heterologous vaccines often result in protection but the birds still shed virulent viruses [27].

Neutralizing antibodies against fusion and hemagglutinin-neuraminidase proteins play an important role in protection against infection [28]. Differences in genotype between vaccine and challenge strains does not hamper the ability of vaccines to protect against disease but viral replication and virus shedding still occurs [29]. However experimental studies have shown that vaccination with genotypic homologous vaccines with challenge virus leads to less virus shedding and therefore reduced virus transmission than heterologous vaccines [29]. It was further demonstrated that significant reduction in virus replication and virus shedding with neutralizing antibodies was more efficient with homologous vaccine strains although higher levels of heterologous antibodies could also reduce transmission [27]. Our present study revealed that neutralizing antibody epitopes on the F protein of our Uganda strains and indeed most of the East Africa isolates were similar to the Lasota vaccine strain which is the most commonly used vaccine strain in Africa and were similar to most genotype V strains both from Africa and elsewhere [11]. The substitutions noted in the epitopes on HN protein and other differences in the aa sequence that could differentiate them from the closely related subgenotype Vd Kenyan strains might suggest possible independent evolution of the Ugandan strains.

The fact that we were able to isolate these highly virulent viruses from apparently healthy-looking birds indicates there was virus replication and shedding that could result in virus transmission and could cause outbreaks. This finding has important bearing on the vaccination strains being used in Africa and provides further data to explore genotype matched vaccines as the most effective strategy to controlling Newcastle disease as has been suggested [27, 28]. Indeed a vaccine study was carried out with South African NDV strains and showed cross-protection of birds with heterologous challenge [30] and a recent ND vaccine efficacy study using Lasota vaccine strain against field strains for West Africa demonstrated protection although the birds still shed virulent viruses [14]. This recent report and the present study indicate that healthy-looking birds may be protected from virulent viruses but continue to shed viruses that may cause disease in naïve populations. The mechanisms behind the survival of velogenic virus in protected birds needs further investigation because it forms part of an important solution to breaking the transmission cycle of these viruses and hence definitive control of ND.

## Conclusions

The present study represents the first Ugandan countrywide genetic study of Newcastle disease viruses demonstrating that highly virulent viruses are replicating and being shed by apparently healthy poultry in live bird markets. They potentially pose a serious risk for outbreaks in the naïve non-commercial poultry populations. It also provides evidence of a new subgenotype Vd of low genetic diversity that is circulating in Uganda, Kenya and Tanzania with implications for the control strategies across the porous borders. While the neutralizing epitopes in these strains showed similarity with the Lasota vaccine strain commonly used for vaccination in the region, the evidence of the replication and shedding of the virulent viruses in apparently healthy birds requires more research. Vaccine strains that could significantly reduce replication and shedding of live virulent viruses would be beneficial.

## Methods

### Sample collection

Tracheal and cloacal swabs were collected from domestic birds from live bird markets in 1500 μl vials of virus transport medium (VTM) across the country with samples collected from representative districts in each region (central, eastern, northern and western) as shown in Figure 1. From each region, districts were randomly selected as follows: central (Masaka, Mukono, and Wakiso), eastern (Abim, Bugiri, Iganga, Kotido, Kumi, Namutumba, and Soroti), northern (Apac, Arua, Gulu, Koboko, Lira, and Nebbi), and western (Kabale, Kabalore, Kasese, and Kisoro). From each of these districts samples were collected from one major live bird market. A total of 1,357 samples were collected with at least 30 samples per selected daily or weekly animal market. The specimens were collected within a 6 months period: from January to June 2011. All samples were transported in a liquid nitrogen dry shipper and stored at −80°C until analyzed.

### Virus isolation and identification

Each field sample was inoculated into 9-10-day old embryonated eggs. Egg inoculation, incubation, candling and virus harvesting were conducted in accordance with the OIE Manual of Standards for diagnostic tests and vaccines [5]. Allantoic fluid was harvested from the eggs and ND virus screened for by hemagglutination (HA) assay. The HA positive samples were subjected to hemagglutination inhibition (HI) tests using reference antisera (PMV-1 monoclonal antibodies or antibodies generated in-house in rabbits against Lasota NDV vaccine strain, boosted at weekly intervals) and 0.5% chicken red blood cells for NDV confirmation. The HA and HI assays were carried out by the microtiter methods as previously described [31]. Virus stocks were stored at −70°C until further use.

### Newcastle disease virus confirmation by RT-PCR

Viruses were further confirmed by PCR before sequencing. Viral RNA was extracted from all samples by using the QIAamp Viral RNA mini kit (Qiagen) according to the manufacturer’s directions. RT-PCR of the extracts was performed by using a Qiagen One-Step RT-PCR kit according to the manufacturer’s instructions, with the following NDV fusion gene primers FOP1: 5′ TACACCTCATCCCAGACAGGGTC 3′ and FOP2: 5′ AGGCAGGGGAAGTGATTTGTGGC 3′ [32]. The 25-μl reaction volume contained 5 μl of 5x RT-PCR buffer, 11 μl of RNAse-free H_2_0, 1 μl of 10 mmol/L dNTPs, 1.5 μl of 10 μmol/L of each primer, 2 μl of 50 mM MgCl_2_, 1 μl of enzyme mix (Taq DNA polymerase and reverse transcriptase), and 2 μl of viral RNA extract. Amplification was carried out in an Applied Biosystems Veriti 96-well thermocycler with a single reverse transcription step of 50°C for 30 min, a denaturation of the RT (95°C) for 15 min, followed by forty cycles with 30-sec denaturation at 95°C, 30 sec of primer annealing at 58°C, 1 min of extension at 72°C, and a final extension for 10 min at 72°C. The samples (including a known positive control) were then separated on a 1% agarose gel with a 100-bp marker. The primers amplified a 532-bp segment of the fusion gene in NDV–positive samples; this product was visualized and documented in a Biorad Gel Doc XR imager.

### Biological characterization

Biological characterization of the isolates was undertaken to classify the pathotypes of the isolates according to OIE [5]. This included the mean death time (MDT) measured for each isolate in embryonated eggs from a specific-pathogen-free source (Lohmann Tierzucht, Cuxhaven, Germany) inoculated by the allantoic cavity route. The MDT was determined by inoculating a standard dose of 1 × 10^6^ 50% egg infectious doses [EID50] into the allantoic cavities of 9- to 11-day-old embryonating specific-pathogen-free eggs. The eggs were incubated at 37°C and candled twice daily (early morning and late afternoon) for 7 days, and the time of embryo mortality was recorded. The MDT for a minimum lethal dose was interpreted as the mean time in hours for the embryo death in the highest dilution at which all eggs died. The isolates were classified as: velogenic viruses if they killed the embryos in fewer than 60 hours; mesogenic if they killed the embryos in 60 to 90 hours; avirulent viruses if they failed to kill the embryos at all. The selected isolates were also tested for virulence in one-day old specific-pathogen-free chicks by intra-cerebral injection of virus. The results were converted into an intra-cerebral pathogenicity index (ICPI) that varied from zero to a maximum of 2.0 for velogenic viruses as described [5]. In addition, the pathotypes were further examined by sequencing of the F0 protein cleavage site motif as described below.

### Sequencing of the fusion (F) and hemagglutinin-neuraminidase (HN) genes

A total of 49 representative isolates were selected for sequencing, among which 19 were selected for full F and HN genes sequencing. In addition to the 19 full F and HN sequences, an extra 10 full F and 19 full HN gene sequences were successfully obtained. For the rest of the 65 isolates, only partial sequencing of the cleavage site was done to determine their amino sequence at this site for pathotyping. To amplify full F and HN genes, cDNA was first generated with random hexamers and revert aid H minus reverse transcriptase (Fisher, Illkirch, France) following the instructions of the manufacturer. PCR was carried out for individual gene fragments with primers described in Table 4, using Taq DNA polymerase (Qiagen). The gene fragments were amplified in an Applied Biosystems GeneAmp PCR system 9700 thermocycler with the following touchdown PCR program: 2 minutes at 95°C, 5 cycles with 30s at 95°C, 30s at 58°C, 1 min at 72°C; 30 cycles with 30s at 95°C, 30s at 56°C, 1 min at 72°C; 5 cycles with 30s at 95°C, 30s at 54°C, 1 min at 72°C; and further extension for 10 min at 72°C. The different fragments were run on a 1% gel and purified by gel purification kits (Qiagen). Sanger sequencing method was carried out on all the segments using the same primers as used for the PCR. Sequencing was performed on a 3130XL Applied Biosystems capillary sequencer at the Plateau de Génomique GeT-Purpan, UDEAR UMR 5165 CNRS/UPS, CHU PURPAN, Toulouse, France.

**Table 4:** **Primer sets used for PCR amplification of F and HN genes of Ugandan NDV isolates**

Gene	Forward primer (5′ to 3′)	Reverse primer (5′ to 3′)	PCR product (bp)
F	NDVF1F^1^: GCAAGATGGGCYCCAAACC	NDVF1R^2^: CATCTTCCCAACTGCCACT	550
	NDVF2F^2^: GACCACTTTACTCACTCCTC	NDVF2R^1^: GTAGGTGGCACGCATATTATT	642
	NDVF3F^2^: CGACTCACAGACTCAACTC	NDVF3R^2^: TATARGTAATRAGRGCRGATG	685
	NDVF4F^2^: GCAAGATRACAACATGTAGRTG	NDVF4R^2^: CTTGGCTAACYGCRCGGTCCAT	709
HN	NDVHN1F^2^: GGCTTCMCAACATCCGTTCTAC	NDVHN1R^2^: GAATGYGAGTGATCTCTGCA	652
	NDVHN2F^2^: CATGAGYRCTACCCAYTACTG	NDVHN2R^2^: GATAGATAAGATGGCYTGCTG	591
	NDVHN3F^2^: GGGTGGCAAAYTACCCAGGAG	NDVHN3R^2^: GTATTGGATATTTCRGCAATGC	768
	NDVHN4F^2^: GCATACACGACATCGACATG	NDVHN4R^2^: CGGTARCCCAGTYAATTTCCA	513

^1^modified from [16].

^2^modified from [32].

### Sequence analysis

The sequences obtained were aligned using Clustal W and edited using Bioedit Software version 5.0.9 [33]. Phylogenetic analysis was performed using the MEGA version 5.05 program [34]. The number of bootstrap replications was set to 500 (for maximum likelihood (ML) analyses) or 1,000 (for Neighbor-Joining (NJ) analyses), and bootstrap values above 50 were labeled on major tree branches for reference. The Ugandan virus strains were clustered on the basis of nucleotides, and only dominant clusters were used to infer phylogenetic relationships. Genotype and subgenotype classification was performed as described by Diel et al., [7]. The analysis included all available full sequences in GenBank or for Figures 2B and 3 all sequences from East Africa in GenBank and the sequence of the reference vaccine strain, Lasota.

### Accession numbers

The complete gene sequences of the isolates analyzed in this study were deposited in GenBank with accession numbers [HG937535 to HG937591]. The isolates with identical sequences were not deposited in the GenBank instead they were represented by one sequence and they have been indicated in the phylogenetic tree legends.
